# Turning Movement Count Data Integration Methods for Intersection Analysis and Traffic Signal Design

**DOI:** 10.3390/s22197111

**Published:** 2022-09-20

**Authors:** Mohammad Shokrolah Shirazi, Hung-Fu Chang, Shahab Tayeb

**Affiliations:** 1 R.B. Annis School of Engineering, University of Indianapolis, Indianapolis, IN 46227, USA; 2 Electrical and Computer Engineering, California State University, Fresno, CA 93740, USA

**Keywords:** turning movement count, vision-based tracking system, SUMO, intersection simulation, intersection analysis

## Abstract

Traffic simulation is widely used for modeling, planning, and analyzing different strategies for traffic control and road development in a cost-efficient manner. In order to perform an intersection simulation, random vehicle trip data are typically applied to an intersection network, making them unrealistic. In this paper, we address this issue by presenting two different methods of incorporating actual turning movement count (TMC) data and comparing their similarity for intersection simulation and analysis. The TMC of three intersections in Las Vegas are estimated separately for one hour using a developed vision-based tracking system and they are incorporated into Simulation of Urban MObility (SUMO) for estimating traffic measurements and traffic signal design. *t*-tests with a 95% confidence interval on the simulation variables demonstrate the importance of using a route-based creation method which injects vehicles into a simulation environment based on the frame-level departure time. The intersection analyses and comparisons are performed based on estimated traffic measurements such as travel time, density, lane density, occupancy, and normalized waiting time. Since the critical edge of each intersection network is identified based on a higher normalized waiting time, new traffic signal designs are suggested based on the actual critical turning movements and improvements in vehicle travel time are achieved to better accommodate the actual traffic demand.

## 1. Introduction

Intersections are defined as a point where different road user (e.g., motorist, bicycle, pedestrian) movements converge, and they have attracted a large amount of attention for network design, management, and planning. Due to the existence of variant road users and conflicting route paths, an appropriate intersection design enhances mobility and safety goals in the public realm. Moreover, traffic signal design is crucial, since it manages different movements, conflict ratios, and road users’ travel time [1]. As a result, monitoring and analysis studies have attracted the attention of transportation engineers and researchers.

For the analysis and design of intersections and their constructive elements (e.g., intersection layout design, traffic light design, etc.), it is critical to monitor traffic flow and extract important traffic data that reflect how well the corresponding design works. For instance, by analyzing the vehicles’ trajectory and amount of delay that vehicles face during their trip, the intersection flow and signal performance are evaluated, and recommendations are considered for optimizing the intersection’s design and signals [2]. Moreover, count data estimation can assist transportation engineers with gauging the capacity of the current design and redesigning the current intersection based on different factors such as exposure, geometry, and signalization to minimize road users’ waiting time while improving their level of safety [3].

A conventional way of turning movement count (TMC) estimation is manual observation and recording of traffic by a human. However, its accuracy is affected in congested scenarios, and it is costly due to its requirement of a human. As a result, some studies [4,5] have aimed to estimate TMC with certain degrees of accuracy. In some cases, such an approach is feasible, as turning movements are predicted in different ways to balance the inbound and outbound traffic. Since these techniques require initial conditions and their outcomes are highly sensitive to the initial assumptions, machine learning techniques and neural networks can help with analyzing the relationship between the approach volumes and predict the corresponding turning movements [6].

As an alternative approach, computer vision can assist with the automatic collection of TMC [7] as long as there is an access to the traffic camera of the target intersection. Even though the accuracy of TMC data is subject to the complexity of the scene (e.g., congestion, occlusion, highly cluttered scenes), it is a good solution for long-term analyses [7]. The following step is to utilize the TMC data, but there are some hurdles that need to be addressed first:

- Supposing that the TMC data is provided by another source (e.g., computer vision, loop detectors), how can this data be useful in providing more insight about the intersection?
- How we can evaluate road users’ statistics regarding the current intersection design, for example, travel time and waiting times of vehicles at the given intersection?
- How we can evaluate the current design of the traffic light, revise its timing based on the flow distribution, and measure its effectiveness?

This paper provides answers to all the aforementioned questions. The basic approach is to incorporate the benefits of the traffic simulators with the actual turning movement data. The actual TMC data will help with minimizing the unrealistic effects of the simulator in addition to having a cost-efficient framework. We propose two different methods of incorporating the TMC data generated from the vision-based tracking system for intersection analysis. We utilized Simulation of Urban MObility (SUMO) [8], which a free-source software that assists transportation engineers and researchers with different evaluations and analyses. The rest of the paper explains the proposed framework in more detail.

The remainder of this article is organized as follows: Section 2 provides insight on the related studies on utilizing traffic simulators for intersection analysis. The two proposed methods of incorporating turning movement count data with developed tracking systems are discussed in Section 3, Section 4 provides the experimental results, and finally, Section 5 concludes the paper.

## 2. Literature Survey

In the realm of intersection analysis using software simulation tools, analysis of the traffic and evaluation of intersection and traffic lights have been addressed by variety of research studies. For instance, Harahap et al. [9] used MATLAB-Simulink for traffic modeling and proposed a queue waiting time model to obtain the average waiting time for each vehicle based on their arrival and the duration time of traffic lights. In [10], a method of controlling traffic signals was presented, based on traffic observations provided by available sensor devices and local communication between traffic lights.

In addition to MATLAB, VISSIM is another common software used for simulation of traffic in different studies [11]. For instance, Arliansyah and Bawono [12,13] evaluated the performance of intersection junctions through measuring some important parameters such as average delay and queue length. Zheng et al. [14] proposed a method for the dynamic use of the left-turn lane for opposite through traffic to improve the efficiency of the signalized intersection. Similarly, the average delay and queue length are the common measurements used to show the efficiency of the proposed method. Yu et al. [13] proposed three methods to evaluate the intersection with different signal timings and find the one that optimizes the objective scenario.

Although VISSIM is an extremely powerful traffic micro-simulation tool that can even be linked to MATLAB, it is not free source, causing limitations in its usage. Alternatively, SUMO has been used by different studies for intersection simulation and analysis [15,16,17]. For instance, Zamborano et al. [15] presented a typical way of incorporating the induction loop measurements to generate an output destination (O/D) matrix of the origin and destination of every trip in the City of Valencia. The work relies on using the DFROUTER [16] tool to generate O/D matrix and it does not specifically address the intersection evaluation by incorporating the matrix directly into SUMO. Elidrissi et al. [17] presented an adaptive traffic light control strategy of intersections using SUMO to reduce the queue time and length.

While there are few recent studies addressing the real traffic scenarios, there is no systematic approach showing how to import real turning movement count (TMC) traffic data for intersection analysis and provide edge-based measurements of intersection networks through estimating crucial traffic measurements such as travel time, lane density, waiting time, and queue length. In this paper, we utilize a vision-based tracking system to provide the 5-min TMC count intervals of three connected intersections in Las Vegas over one hour. The collected data are followed by two different methods of incorporating TMC data into SUMO and comparing the similarity of simulation measurements (e.g., wait before insertion time, latest travel time, mean speed, and mean relative speed) using *t*-tests. Due to frame-based resolution of the route creation method for incorporating TMC flows into SUMO, experimental data from this method are further analyzed to provide comprehensive analysis on the simulated intersections. More insights about different edges of the observed intersection are provided using various critical measurements such as travel time, normalized waiting time, lane density, occupancy, and queue length. Finally, the intersection edges with the highest queue length are identified, and a new design for traffic signals is suggested based on actual critical turning movements to reduce the average vehicles’ travel time. The remainder of the paper explains the integrated methods and the extraction of measurements in more detail.

## 3. Intersection Simulation System Framework

**Figure 1:**
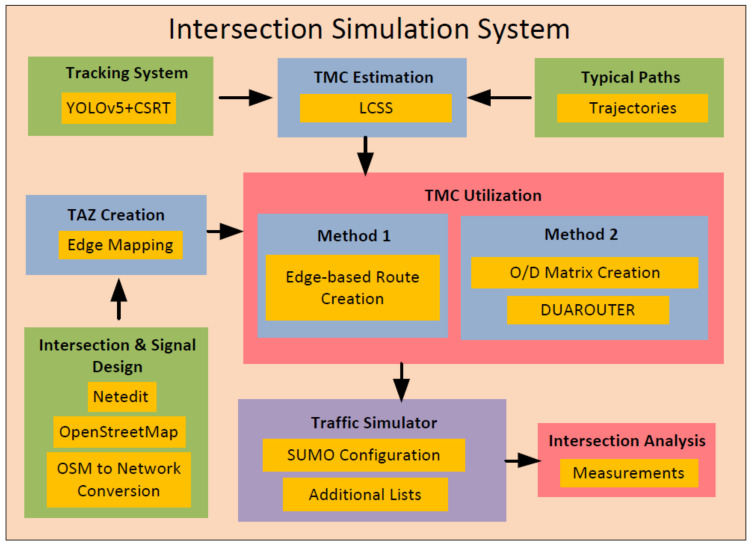
The proposed framework for intersection analysis.

The simulation system framework, which is conducted until intersection analysis, is presented in Figure 1. A variety of components and steps are required to extract TMC data and incorporate them through the two proposed methods within a SUMO simulation framework and finally analyze the intersection based on the extracted traffic measurements.

### 3.1. Tracking System

The tracking system utilizes computer vision techniques to provide labels for individual road users using traffic cameras. The main reason for using computer vision techniques is to provide the count data for a long time period in a cost-efficient way. Since intersections host a mix of road users (e.g., vehicles, bicycles) with varied motion patterns, state-of-the-art techniques are required to provide detection of each road user over the sequence of frames. As an outcome, You Only Look Once (YOLO) architecture is a reasonable choice since it provides predictions in one pass of an image, resulting in faster detection in comparison with other convolutional neural network-based techniques such as faster RCNN [18]. We utilized YOLOv5 [19] for this purpose due to its higher speed and accuracy as compared to former YOLO versions such as YOLOv4 [20] and YOLOv3 [21].

The detection system generates a detection list used to initialize the tracking list. During the tracking phase, detection and tracking lists are updated and the tracking list will maintain the trajectory of each individual road user during their tracking lifetime.

For the first frame, the trajectory list (TL) is initialized with the detection list (DL), and for other frames, three main methods, namely update(), add(), and remove() are sequentially run for every frame captured from the input stream. The update() method performs discriminative correlation filter tracking (i.e., CSRT() [22]) for each tracked component in the TL. The CSRT method is utilized, since it has shown a high performance in accuracy as well as robustness in tracking for variety of video settings.

The IoU ratio is used as the criterion to determine whether there is a good detection in the DL list (see Equation (1)) or not. A bipartite graph is constructed to match detections to tracks in a greedy fashion. A detection is used by update() when it has a high overlap with an existing track. Then, the intersection over union (Equation (1)) IoU>0.50%, the average of bounding boxes coordinates between track and detection, is estimated and added to the trajectory list (TL). If no good detection is found for a track in TL, update() will estimate the next bounding box coordinates for a few frames before the track’s removal from the active list of tracking objects.

(1) $$IoU\left(TL,DL\right)=\frac{TL\cap DL}{TL\cup DL}$$

When a detection does not match the existing tracks in the track list during the association process, a new track is created. This task is performed by leveraging the IOU ratio within the add() method. If an existing track does not find a detection for a sequence of three frames, it is marked for deletion through the remove() method.

### 3.2. Turning Movement Count Estimation

**Figure 2:**
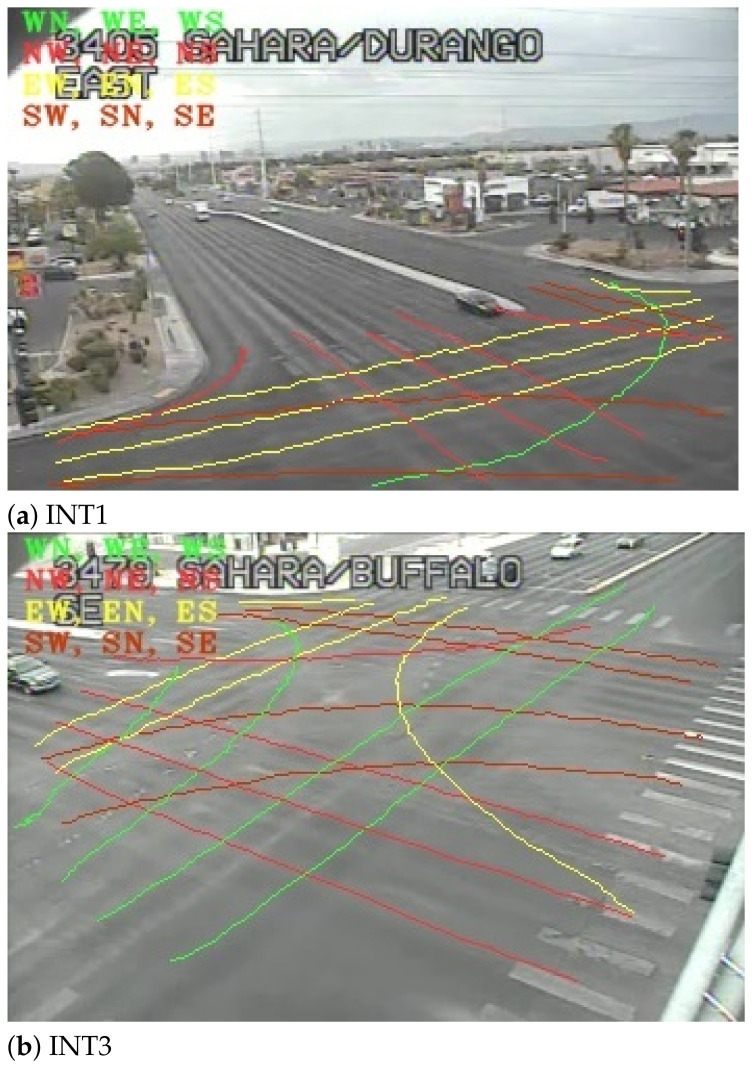
Typical paths.

The turning movement and pedestrian crossing counts were conventionally acquired by technicians who manually recorded the count number of vehicles and pedestrians going each direction during daily peak hours. This becomes tedious and costly for turning movement counts that needs count records from different directions that occur concurrently. The proposed system automatically recognizes the vehicle and pedestrian movement patterns using the longest common sub-sequence (LCSS) method with the modeled typical paths of each intersection (see Figure 2). LCSS is used as a path comparison tool, due to its effectiveness against noise and outliers and its good performance for two unequal paths [23].

### 3.3. Typical Paths

An essential step in determining the turning movement count is to provide some reference trajectories for comparison, called typical paths. The typical paths consist of (x,y) sequences as predefined common moving patterns. The observed trajectory points of each road user are compared against each typical path using the LCSS method and the path that has the highest similarity score based on a number of matched (x,y) points [23] is returned. For instance, typical paths are sketched in Figure 2 for two different intersections. The paths are color coded based on their starting zone regions (e.g., green for west-to-east (WE), west-to-north (WN), and west-to-south (WS) paths). As a matter of fact, a maximum of 12 typical paths could exist for a typical intersection.

### 3.4. Intersection and Signal Design

The main objective for studying intersections and their research directions is the identification of the data collection system requirements and the selection criteria for the target intersection. Since our objective is to obtain TMC data estimation using the developed vision-based tracking system, we need to identify the intersections that have accessible traffic cameras. Moreover, intersection layouts corresponding to monitored intersections are required to be designed for simulation purposes. In this work, the NETEDIT tool, a road network editor for road traffic simulation in SUMO, is utilized. This step reflects the determination of the actual number of lanes for each direction of the intersection that can be verified though scene investigation using traffic cameras as well as Google Maps.

After creating the nodes and edges which contain the actual number of lanes for each observed intersection, traffic lights need to be designed and applied at a junction. The traffic signal states are defined for each moving directions using characters such as G (green), g (green), y (yellow), and r (red). While G shows the priority, g causes vehicles to stop if another vehicle uses a higher-prioritized flow stream. Vehicles must stop if they face r, and y causes them to decelerate if they are far away from the junction or otherwise pass through the junction. More details about the actual design of traffic signal lights are stated in Section 4.3.

### 3.5. Traffic Assignment Zone Creation

In order to analyze traffic from a network edge to another edge, traffic assignment zones (TAZs) are conventionally generated to provide new labels for the edges as information identifiers. This will be applied into the network file to demonstrate the route paths of the simulated vehicles.

**Figure 3:**
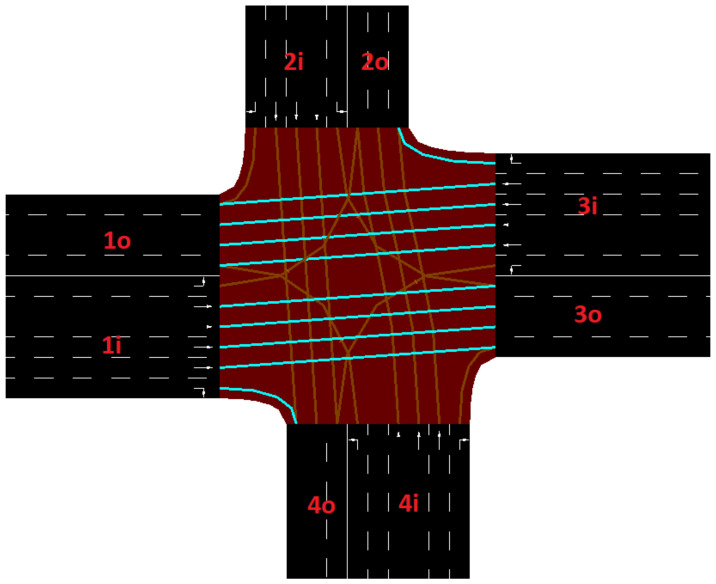
Label assignments for network edges (INT1).

Figure 3 shows a typical mapping of an observed intersection with new labels for each edge. West (W), north (N), east (E), and south (S) zones have been arbitrary labeled by 1, 2, 3, and 4 and i and o indicate input and output flow directions for the junction, respectively.

### 3.6. TMC Utilization

Two methods are presented in this paper to incorporate TMC data of the vision-based system for the simulation: O/D (output/destination) matrix route creation and edge-based route creation. More details for each method are as follows:

#### 3.6.1. Method 1: O/D Matrix-Based Route Creation

In this method, TMC data are organized in the form of an output/destination (O/D) matrix where each row represents the outward and inward flow edges and the amount of corresponding count data. This matrix provides SUMO with vehicle trips from each flow direction on the intersection network edge. Due to the creation of the TAZ, the O/D matrix can be simply arranged based on the new labels referring to the edges and TMC data that have already been defined within the TAZ file. For example, a row of 1i, 3o, 15 implies that a total of 15 vehicles took the WBT route in their travel path during the simulation time.

Due to existence of the trip demand model for every flow direction as well as the intersection network, the DUAROUTER [8] tool generates the routes of the vehicles based on the shortest path computation for the entire simulation time. Although the generated route file contains the vehicles’ flows between different TAZs according to the O/D matrix, it requires a large amount of computational hardware resources (e.g., RAM, CPU) because it looks for the shortest route between two defined points on the map [24].

#### 3.6.2. Method 2: Edge-Based Route Creation

Edge-based route creation refers to creating the route paths directly as an output of the vision-based tracking system. Since the tracking system can determine the turning movement count with prior knowledge of the departing frame number, the turning movement routes can be created according to departure time based on the TAZ. This method does not follow the shortest path algorithm and reflects actual traffic based on a higher time resolution, since each vehicle is labeled based on departed time frame for all movement directions.

### 3.7. Traffic Simulator

In order to set up the simulation, the generated network file, route file, and simulation time with a list of additional files to address traffic measurements as output are prepared and identified for the traffic simulator. The traffic simulator receives the configuration file and conducts the simulation process by injecting the vehicles according to the route file into the intersection network. The output information regarding the simulated vehicle statistics as well as the edge-based traffic measurements is generated after completing the simulation experiments.

### 3.8. Intersection Analysis

Intersection analysis is a data analysis process that utilizes extracted measurements and their visualization to obtain more insight regarding road user movement patterns and traffic management facilities. For instance, the vehicle waiting time and speed are two crucial measurements for addressing the efficiency of the traffic signals and intersection capacity. Here are some important measurements that we have utilized in this work for intersection analysis:

- Travel time (TT): Time in seconds that vehicles require to pass the edge/lane, which is calculated based on the mean speed for the front of the vehicles.
- Density (DE): Number of vehicles per kilometer (#veh/km) for the edge of the intersection network.
- Lane density (LD): The density divided by the number of lanes, i.e., the vehicle density on the edge per lane (#veh/km/lane).
- Occupancy (OC): Ratio of vehicles to the distance, presented in percentage. A value of 100 indicates that vehicles are placed bumper to bumper on the whole edge with a minimum gap of zero.
- Normalized waiting time (NW): The accumulated number of seconds that vehicles were considered halting (speed < 0.1 m/s) over the total number of vehicles on the edge.

## 4. Experimental Results

### 4.1. Data Collection System Evaluation

The evaluation results for the vision-based detection system show how well the existing YOLO architecture can deal with the detection of low-resolution and small road users. The precision and recall metrics for evaluation of the detection system are defined in Equation (2).

(2) $$Precision=\frac{tp}{tp+fp},\;Recall=\frac{tp}{tp+fn}$$

True positive (tp) is the number of correctly detected objects, false positive (fp) is the number of falsely detected objects, and false negative (fn) is the number of missed detections for each object category.

Table 1 shows the evaluation results of the tracking system in detecting vehicles for the three intersections with the camera numbers 2101, 2203, and 2175. The three intersection videos were annotated with bounding box locations of the existing vehicles for 1000 frames each. The evaluation results indicate that off-the-shelf YOLOv3 architecture provides slightly better precision (0.96) and recall (0.70) values in comparison to YOLOv5 for small vehicles. In this work, YOLOv5 is utilized since it provides a faster detection speed in comparison with its former variants [25].

### 4.2. Target Selection, Preparation and Setup

**Figure 4:**
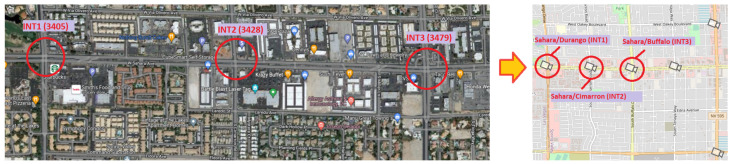
Three intersections with accessible traffic cameras targeted for automated monitoring and analysis.

Three connected intersections in Las Vegas that have public closed-circuit television cameras for traffic monitoring were selected for our experiments. The cameras are provided by the Freeway and Arterial System of Transportation (FAST) as a division of the Regional Transportation Commission of Southern Nevada (RTC). Figure 4 shows the geographical location of the three intersections with the corresponding cameras.

The vision-based tracking system for remotely monitoring the cameras was implemented with Python 3.6 and OpenCV 4.5.2 and it was run on a dedicated high-end PC with a quad-core Intel i7-7700 3.6 GHz processor, 16 GB RAM, and an Nvidia Geforce GTX 1070 GPU with 8 GB of total RAM and 2048 CUDA cores. Additional Python scripts were developed for trajectory collection of road users and preliminary analysis such as estimates of turning movement counts. Eclipse SUMO 1.13.0 and its package tools (e.g., NETEDIT, DUAROUTER) were used for intersection simulation and further analysis.

In order to simulate the intersections with appropriate settings, the actual number of lanes need to be designed for each edge of the intersection network. Table 2 shows the number of lanes created for each edge of the three intersection networks addressed for traffic monitoring and analysis. For inward flow directions to the junction, INT1 has the highest number of lanes: six for the west, east, and south zones (i.e., 1i, 3i, 4i). As a matter of fact, INT2 has the lowest inward and outward flow capacities, since two lanes exist on outward flow direction edges (e.g., 2o, 4o) and three lanes exist on inward flow direction edges (e.g., 2i, 4i).

### 4.3. Basic Signal Design

**Figure 5:**
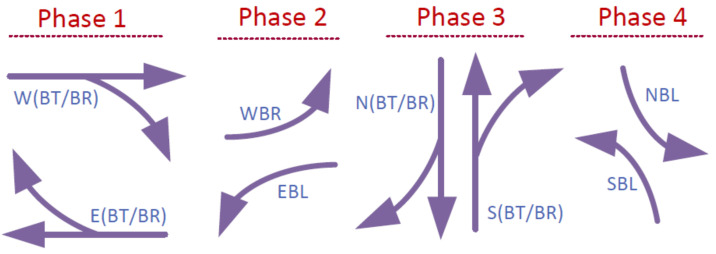
Four major signal phases. Left turns of phases 2 and 4 are protected and there are permissive left turns associated with phases 1 and 3.

After creating the intersection network through creating nodes, edges, and revising the number of lanes on each edge, a traffic light is placed at the junctions of the edges to manage different flow directions. The default signal design consists of four major phases, where two phases are branch through and branch right from the west/east and north/south zones and two are protected branch lefts. Figure 5 shows the graphical representation of the phases of basic traffic light design for intersections.

The basic traffic signal is designed to provide a fair amount of time dedicated to each flow direction. While the main flow stream direction (i.e., go through) has a higher phase time (i.e., 33 s), it overlaps with the flow of the opposite direction to maximize the occupancy since they do not have conflicts. While the vehicles with a flow in the left-branching direction are permissive and should yield to the other conflicting income flow directions, they have a protected phase of 6 s to resolve the starvation issue. Since each major signal phase is followed by a yellow traffic light for a duration of 3 s, eight total signal phases will create a traffic light cycle to manage the traffic of all turning flows at a junction. As a result, the basic signal will have a 90-s cycle time where the four major phases one, two, three, and four constitute 33.6%, 6.6%, 33.6%, and 6.6% of the duty cycle, respectively, and each yellow signal is 3.3% of the duty cycle.

### 4.4. Turning Movement Count Estimation

The three intersections (INT1–3) were monitored separately using the developed vision-based tracking system on July 18th of 2022 from 7:00–8:00 am and their turning movement counts were estimated with 5-min intervals using typical paths and the LCSS method for route path similarity comparison.

**Figure 6:**
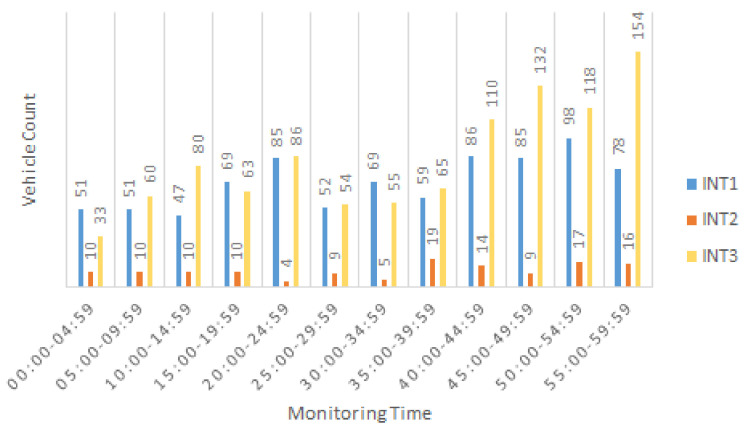
Branch-left (BL) count estimation in 5-min intervals.

Figure 6 shows the branch-left counts of each intersection with 5-min intervals. The highest left-turn count belonged to INT3 at 7:55–8:00 a.m., and INT1 had a left-turn count close to that of INT3 at some time points, such as 7:20–7:25 a.m. (e.g., 85 (INT1) and 86 (INT3)). The left-turn count for INT2 was the lowest, which implies that reducing the corresponding protected green light time would result in mobility improvement.

**Figure 7:**
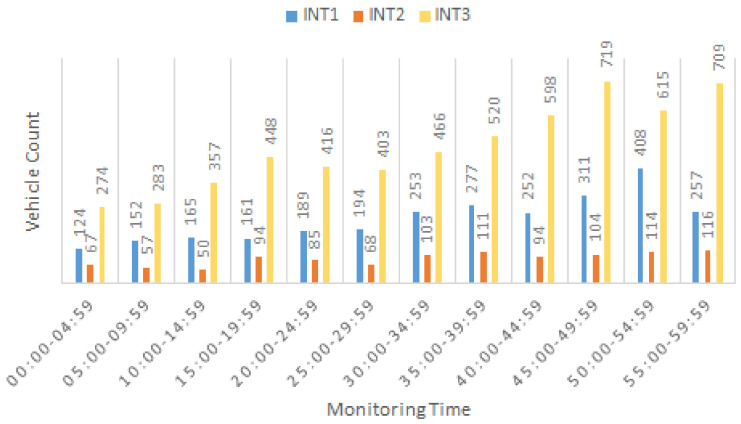
Branch-thru (BT) count estimation in 5-min intervals.

Figure 7 depicts a high volume of vehicles moving in straight directions for all three intersections. Evidently, INT3 had the highest number of straight-moving vehicles for all monitoring intervals and INT1 and INT2 had more similar count patterns. The highest number of branch-through vehicles for INT2 was in the period from 7:50–7:55, making this time the most crowded time for all three intersections taken together. Similarly, INT2 had the lowest number of vehicles going straight.

**Figure 8:**
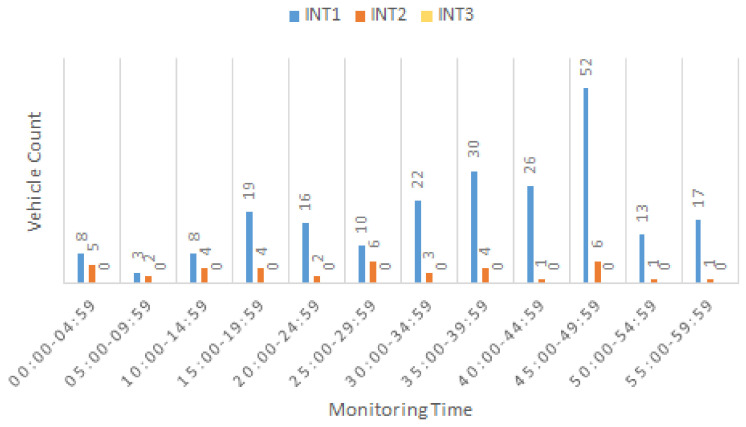
Branch-right (BR) count estimation in 5-min intervals.

Figure 8 shows that INT1 had the highest volume of right-turning vehicles, as we expected. Due to the limited field of view for the existing camera monitoring INT3, no right turns were captured for this intersection, and INT2 had the lowest count of right-branching vehicles. The highest right-turn count was observed at 7:45–7:50 for INT1. The classification of counts for different turns at each intersection implies that while the highest signal phase time should be considered for straight movements, left-turning vehicles also require separate exclusive green phases (i.e., G) as a protected phase. However, right-turning vehicles may take a permissive green light (i.e., g), due to lowest number of captured counts in this flow direction.

### 4.5. TMC Data Integration Methods Comparison

The estimated turning movement counts are utilized for calibration of each intersection simulation using two proposed methods: O/D matrix-based route creation (M1) and edge-based route creation (M2). As we stated in Section 3.6, M1 relies on randomly generated route paths within a one-hour simulation with respect to the total number of TMCs, while M2 maintains the time order by recording the second for each turning flow direction. While M1 generates the O/D matrix-based routes based on the total estimated TMC counts for 1 h of monitoring time as the output of the tracking system, it does not keep the departure time second for each moving direction. This shows the importance of the tracking system in providing appropriate file formats for importing into SUMO as configuration files for the simulator.

In order to gauge the similarity of the two methods, the simulation of each intersection for the one hour of monitoring time was performed and the simulation-related measurements were estimated. The simulation-wide measurements provide statistics on the time that vehicles needed to complete their routes and the number of vehicles’ states in the simulation, such as loaded, inserted, running, waiting to be inserted, and reached their destination. The simulation measurement results that we utilized for comparison of the two methods are as follows:

- Wait to insertion time (WIT): The mean time that all vehicles until now and within the reported time step had to wait before being inserted.
- Mean latest travel time (MLT): The mean travel time of all vehicles that have left the simulation within the previous and reported times.
- Mean speed (MSP): The mean speed over all vehicles in the network.
- Mean relative speed (MSR): The mean speed of all vehicles in the network relative to the speed limit.

*t*-tests were performed to find the true difference between the two groups of measurements using the simulation output for each method in 3600 time step observations. The two-tailed tests for unequal variance for each of the two groups of extracted simulation measurements are shown in Table 3. The test results with 95% confidence intervals show a significant difference for all measurement values except MSP and MSR for INT1. Although the average and standard deviation of MLT are close for INT1, there is a significant difference between the observed data of the two different methods. As we expected, the average and standard deviations of MSP and MSR are closer in comparison to those of WIT and MLT. Method 2 provides a lower mean travel time but a higher WIT due to not optimizing the shortest path as result of the use of the DUAROUTER tool for all intersections. Additionally, the higher mean latest travel time reflects a lower mean speed, as we observe INT1 has a higher average MLT than INT3 but a lower MSP.

Based on the comparison between the two proposed methods, there is a significant difference in the simulated measurements if the start time order of turning movements (in seconds) is not respected, even though the total count might be same for the entire hour. So, Method 2 is preferred since it reflects the correct timing labels for each travel path movement and also provides a lower mean travel time for each injected vehicle into the simulation environment. Consequently, Method 2 (M2) is deployed in the rest of this paper for intersection simulation and further analysis.

### 4.6. Intersection Analysis

#### 4.6.1. Measurement-Based Intersection Comparison

**Figure 9:**
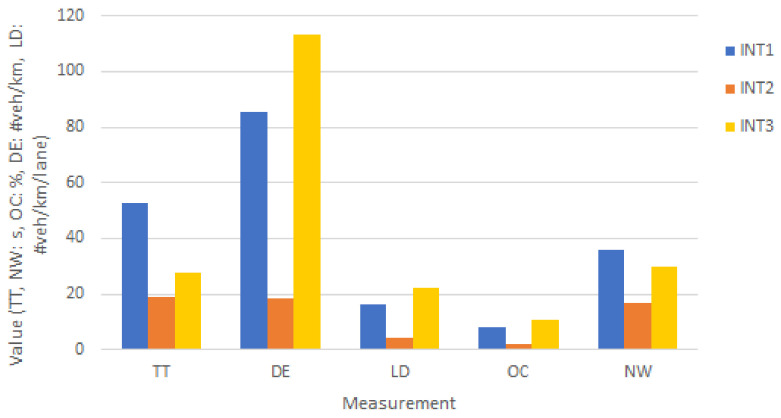
The comparison of simulated intersections (TT: travel time, DE: density, LD: lane density, OC: occupancy, NW: normalized waiting time).

As result of simulating three different intersections, traffic measurements are generated for each edge of the intersection. Figure 9 shows the intersection comparison based on extracted edge-based measurements such as travel time (TT), density (DE), lane density (LD), occupancy and normalized waiting time (NWT) (see Section 3.8).

The highest density and occupancy belong to INT3 and INT2, respectively, which are correlated with the higher turning movement count data shown in Section 4.4. However, the normalized waiting time (NW) of INT1 is higher than that of INT3 since the waiting time is divided over a higher number of departed vehicles to calculate the normalized values. As a result of this comparison, INT1 and INT3 need to be considered for traffic signal optimization due to the higher normalized waiting time observed over same hour for the three intersections.

#### 4.6.2. Queue Length

The normalized waiting time was calculated for each edge of the three intersections, and edge 1i demonstrated the highest waiting time value. This is due to the high volume of vehicles moving from the west zone to other directions and left-turning vehicles that cause obstacles in front of the following vehicles. Motivated by this issue, we extracted the queue length on edge 1i to compare this important parameter’s difference between the intersections.

**Figure 10:**
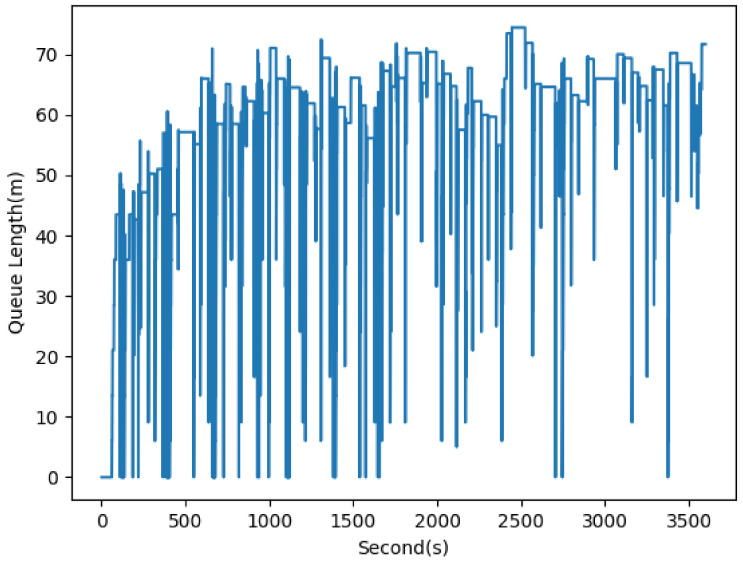
Queue length estimation for edge 1i of INT1 (AVG: 57.59 m, STD: 16.36 m).

Figure 10 shows the queue length results on edge 1i, which demonstrated the highest waiting time for the entire simulation duration. A high queue length of 70 m was observed at some simulation time steps (e.g., 2500 s) and there are few time steps at which the queue length reached zero. This edge has an average queue length of 57.59 m, which is relatively high in comparison to the other intersections.

**Figure 11:**
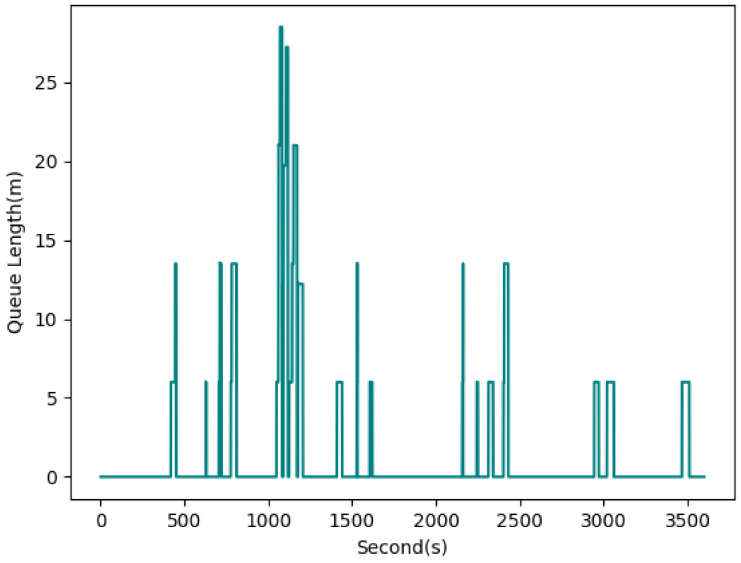
Queue length estimation for edge 1i of INT2 (AVG: 1.32 m, STD: 4.14 m).

Figure 11 shows the queue length estimate for INT2, which is 0 for a large portion of the simulation time. The reason for this observation is the lower number of vehicles traveling through this intersection, as we noticed earlier with the low turning movement count data. Interestingly, the standard deviation is relatively high in comparison with the average, shown as peaks at some time points. For example, a peak queue length of higher than 25 m can be observed after 1000 s of simulation time.

**Figure 12:**
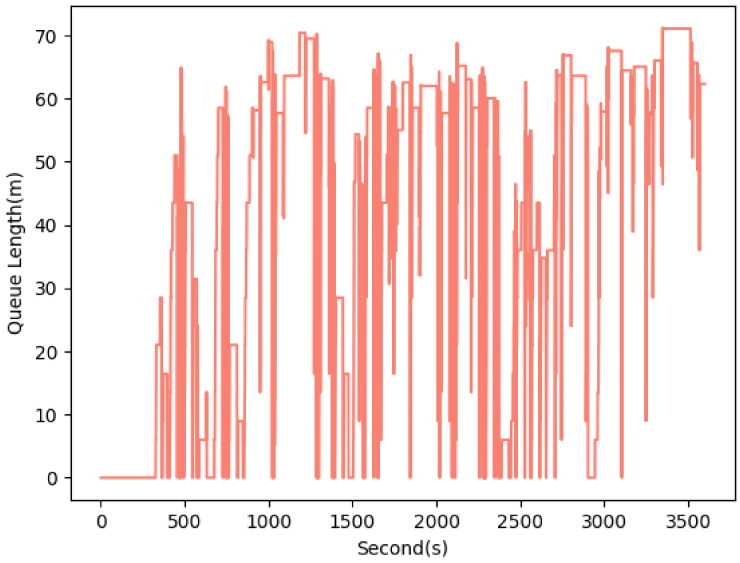
Queue length estimation for edge 1i of INT3 (AVG: 42.15 m, STD: 25.74 m).

Similarly, Figure 12 presents the queue length for INT3, which reaches approximately 70 at some time points (e.g., 3400 s). Comparison of the queue length shows that INT1 and INT3 require a revisit of the traffic signal timings, especially for the straight-through directions due to the high queue length observed on edge 1i. Due to the higher standard deviation of INT2, a dynamic setting for this traffic signal is suggested as a result of the queue length experiments.

#### 4.6.3. Traffic Signal Design Improvement

Based on observation from the queue length on edge 1i, we re-evaluated each intersection to improve the vehicles’ travel times and waiting times. Since the actual turning movement count was provided by the vision-based tracking system during the monitoring time, the traffic phases can be designed based on the critical turning movement which is based on the highest count of movement in the same phase. Table 4 shows the critical movements that are considered in the signal treatment and their distributions.

The results shows that INT3 and INT1 hosted the highest number of vehicles based on their collected turning movement count data. This is supported by the highest density value in Figure 9 for INT3. Interestingly, higher travel time (TT) and normalized waiting time (NW) were observed for INT1 (see Figure 9), increasing the priority of INT1 for redesign of its traffic signal lights.

**Figure 13:**
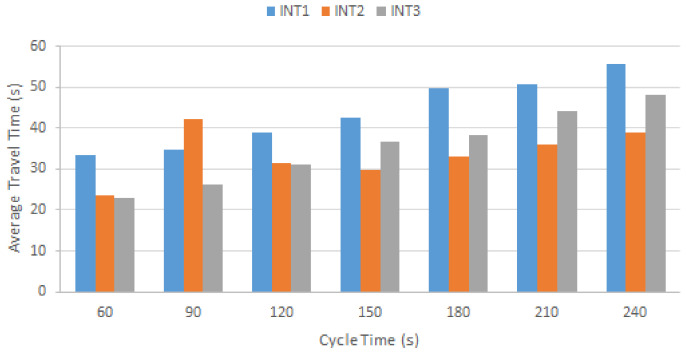
Vehicle average travel time after signal treatment.

Figure 13 shows the average travel time after redesigning the traffic lights based on the turning movement distributions presented in Table 4. Even though the distribution time is adjusted based on cycle length, increasing the cycle length results in a higher average travel time for all three intersections.

Table 5 shows the improvements for INT1, INT3 after redesigning the traffic signal timing based on critical turning movements and their distribution. The estimated average vehicles’ travel time with the default signal design (see Section 4.3) were 52.89, 18.75, and 27.50 s for INT1, INT2, and INT3, respectively. The results of a comparison with the estimated data depicted in Figure 13 implies the highest improvement was granted by the 60-s signal cycle for all intersections except INT2. As we noted earlier in Section 4.6.1, INT2 has the lowest number of turning movement counts, which excludes this intersection from traffic signal treatment. Moreover, three major phases have small and similar turning movements, which resulted in a very short green time since the majority of the signal cycle is dedicated to phase one: 75% for the W/E (BT/BR) directions. This causes a starvation issue and a long waiting time for vehicles in the other three directions. As an outcome, while designing traffic signals based on turning movement counts are preferred, we need to consider the minimum phase time of other directions, which should not be less than a reasonable amount to avoid the starvation issue.

## 5. Conclusions

In this paper, two different methods of simulating intersections and performing analyses with real turning movement data are presented. Three intersections at Las Vegas are studied due to accessibility of their traffic cameras and the estimated turning movement count of 1-h periods were imported into SUMO using two methods of O/D matrix-based and edge-based route creations. As a result of *t*-tests on simulation variables of these two methods, edge-based route creation method is deployed further for intersection analysis and important traffic measurements such as travel time, density, lane density, occupancy, and normalized waiting times are estimated for each intersection. The experimental results manifest the highest waiting time on west bound edges on all three intersections due to high volume count of vehicles departing from this and high portion of them intend to turn left. Further analysis on queue length estimate of this edge shows a necessity of re-designing the traffic signal for INT1, and INT3 to better accommodate the high volume of vehicles waiting at specific time intervals. Subsequently, new design for traffic signals is suggested and evaluated based on critical turning movement data to minimize vehicles waiting time and their travel time. This work can be further used as direction for traffic engineers and researchers to calibrate their traffic simulators with actual turning movement count data to better analyze the requirements of their current intersections design and improve their traffic signal design in smart cities.

## Figures and Tables

**Table 1:** Detection system evaluation for intersection monitoring (Pre: precision, Rec: recall).

Intersection	YOLOv3		YOLOv4		YOLOv5	
	Pre	Rec	Pre	Rec	Pre	Rec
INT 2101	0.92	0.55	0.81	0.61	0.87	0.67
INT 2203	0.98	0.76	0.83	0.91	0.91	0.79
INT 2175	0.99	0.80	0.83	0.86	0.94	0.72
AVG	0.96	0.70	0.82	0.79	0.91	0.73

**Table 2:** Number of lanes for each edge of intersection network.

Intersection	West		North		East		South	
	1i	1o	2i	2o	3i	3o	4i	4o
INT1 (3405)	6	4	5	3	6	4	6	3
INT2 (3428)	5	4	3	2	5	4	3	2
INT3 (3479)	6	4	5	3	6	4	5	3

**Table 3:** Means and standard deviations of the simulation measurements. (MEAS: measurement, M1: Method 1, M2, Method 2, WIT: wait to insertion time, MLT: mean latest travel time, MSP: mean speed, MSR: mean relative speed).

Intersection	MEAS	M1		M2		df	p
		AVG	STD	AVG	STD		
INT1 (3405)	WIT	25.73	18.21	32.17	22.95	6845	0.00
	MLT	55.18	9.81	54.57	8.59	7074	0.00
	MSP	2.96	1.38	2.93	1.36	7195	0.30
	MSR	0.21	0.1	0.21	0.09	7186	0.34
INT2 (3428)	WIT	0.99	0.03	6.32	2.00	3601	0.00
	MLT	34.24	3.85	4.83	3.48	7196	0.00
	MSP	6.17	3.85	4.83	3.48	7125	0.00
	MSR	0.44	0.26	0.34	0.26	7191	0.00
INT3 (3479)	WIT	178.51	119.86	102.29	64.62	5528	0.00
	MLT	60.60	9.19	49.24	8.28	7121	0.00
	MSP	2.97	1.32	3.35	1.55	7015	0.00
	MSR	0.21	0.09	0.24	0.11	7056	0.00

**Table 4:** Critical turning movements and their distribution for traffic signal design and treatment.

Intersection	W/E (BT/BR)	W/E (BL)	N/S (BR/BT)	N/S (BL)
INT1	677.5	505	485.5	233
	0.356	0.265	0.255	0.122
INT2	372	49	32	40
	0.754	0.099	0.064	0.081
INT3	738.5	441	1160	318
	0.277	0.165	0.436	0.119

**Table 5:** The comparison of traffic measurements (i.e., travel time (TT), normalized waiting time (NWT)) before and after signal design and treatment.

Intersection	INT1		INT2		INT3	
	TT	NW	TT	NW	TT	NW
Default Signal	52.89	35.85	18.75	8.46	27.5	14.98
After Signal Design	33.28	18.91	23.47	12.8	22.9	10.61

## Data Availability

The data presented in this study are available on request from the corresponding author.
